# Identification of a Small Supernumerary Marker Chromosome in a Turner Syndrome Patient with Karyotype mos 46,X,+mar/45,X

**DOI:** 10.3390/genes14020253

**Published:** 2023-01-18

**Authors:** María Teresa Alejandra González-Rodríguez, Sinhue Alejandro Brukman-Jiménez, Idalid Cuero-Quezada, Jorge Román Corona-Rivera, Alfredo Corona-Rivera, Graciela Serafín-Saucedo, Liuba M. Aguirre-Salas, Lucina Bobadilla-Morales

**Affiliations:** 1 Human Genetics PhD Program, Department of Molecular Biology and Genomics, Centro Universitario de Ciencias de la Salud, Universidad de Guadalajara, Guadalajara 44340, Mexico; 2 Human Genetics Institute “Dr. Enrique Corona Rivera”, Department of Molecular Biology and Genomics, Centro Universitario de Ciencias de la Salud, Universidad de Guadalajara, Guadalajara 44340, Mexico; 3 Cytogenetics Unit, Hospital Civil de Guadalajara Dr. Juan I. Menchaca, Guadalajara 44340, Mexico; 4 Center for Registry and Research in Congenital Anomalies (CRIAC), Service of Genetics, Pediatrics Division, Hospital Civil de Guadalajara Dr. Juan I. Menchaca, Guadalajara 44340, Mexico; 5 Service of Pediatric Endocrinology, Pediatrics Division, Hospital Civil de Guadalajara Dr. Juan I. Menchaca, Guadalajara 44340, Mexico

**Keywords:** small supernumerary marker chromosome, turner syndrome, intellectual disability

## Abstract

Turner Syndrome is characterized by a normal X chromosome and the partial or complete absence of a second sexual chromosome. Small supernumerary marker chromosomes are present in 6.6% of these patients. Because of the wide range of Turner syndrome karyotypes, it is difficult to establish a relationship with the phenotype of the patients. We present the case of a female patient with Turner syndrome, insulin resistance, type 2 diabetes, and intellectual disability. The karyotype revealed the presence of mosaicism with a monosomy X cell line and a second line with a small marker chromosome. FISH of two different tissues was used to identify the marker chromosome with probes for X and Y centromeres. Both tissues presented mosaicism for a two X chromosome signal, differing in the percentage of the monosomy X cell percentage. Comparative genomic hybridization with the CytoScan^TM^HD assay was performed in genomic DNA from peripheral blood, allowing us to determine the size and breakage points of the small marker chromosome. The patient presents a phenotype that combines classic Turner syndrome features and unlikely ones as intellectual disability. The size, implicated genes, and degree of inactivation of the X chromosome influence the broad spectrum of phenotypes resulting from these chromosomes.

## 1. Introduction

Turner Syndrome (TS) is a chromosomal disorder characterized by a structurally normal X chromosome and the partial or complete absence of a second sex chromosome. The incidence is 1:2500 newborn girls, and the estimated prevalence is 25–50 cases per 100,000 women. Monosomy X (45,X) is the classical karyotype in TS, present in 40–50% of patients. Thirty percent of cases present karyotypes with structural aberrations of the X chromosome, the most frequent being deletions, isochromosomes, and ring chromosomes [1].

TS is a heterogeneous chromosomal disorder in which phenotype varies significantly among patients. The classic clinical findings are short stature, ovarian dysgenesis, and premature ovarian failure [2]. The manifestations become more severe as the chromosome X material decreases [3,4]. Patients with pure monosomy X tend to be diagnosed at an earlier age and present the characteristic TS phenotype: infancy acral lymphedema, low-set hairline, short and webbed neck, shield chest, wide-spaced nipples, *cubitus valgus,* multiple *nevi,* delayed puberty, and amenorrhea [2,5,6].

Small supernumerary marker chromosomes (sSMC) are structurally abnormal chromosomes that cannot be identified or characterized by karyotyping with GTG banding and are smaller than chromosome 20 of the same metaphase spread [7]. Among TS cases, 6.6% carry an sSMC (sSMC^T^) with an approximated frequency of 1 marker chromosome per 100,000 newborn patients [7,8]. The sSMC database records 562 cases of sSMC^T^ up to January 2023. Most frequently, these markers derived from the Y chromosome (50.4%), followed by the X chromosome (48.9%), with only four case reports from autosome-derived marker chromosomes to date [9].

sSMC^T^ presents shapes as isodicentric/inverted duplicated, ring, or centric minute. Ring formation is predominant in markers derived from the X chromosome through either double terminal deletions of both arms of the chromosome or by fusion of the telomeres without deletion [10]. sSMC^T^ were present in 0.06% of cases of developmental delay (DD) or ID in the case series reported by Liehr in 2007 [7]. It has been estimated that 0.3% of neurodevelopmental diseases are associated with mosaicism of the X chromosome and small supernumerary marker chromosome [11]. ID and DD are frequent findings of ring or marker chromosomes derived from the X with failure to be inactivated due to the deletion or dysfunction of the *XIST* gene [10].

The variety of karyotypes present in TS, the origins and sizes of sSMC^T^, and the changing degree of mosaicism, even between different tissues of the same individual, challenge the assessment of a direct relationship with the phenotype of the patients. Therefore, it is crucial to establish the origin and morphology of sSMC^T^ as well as the size and genes that it contains. This case report describes a patient with TS and an sSMC^T^ derived from the X chromosome characterized by a cytogenomic approach.

## 2. Materials and Method

### 2.1. Clinical Study

A multidisciplinary team that included pediatric specialists, such as an endocrinologist, cardiologist, clinical geneticist, and neuropsychologist, evaluated the patient. Informed Consent was obtained from the parents, as well as an informed assessment from the patient.

### 2.2. Karyotype and FISH

Peripheral blood lymphocyte cultures were stimulated with phytohemagglutinin to obtain metaphase chromosomes. GTG banding was performed according to standard procedures [12]. The chromosomes were analyzed and karyotyped following the International System for Human Cytogenetic Nomenclature (ISCN). FISH was performed in interphase cell nuclei obtained from peripheral blood and buccal mucosa, according to the manufacturer’s protocol with the AneuVysion Multicolor DNA probe kit (Abbot, Chicago, IL, USA), which combines specific probes for the regions D18Z1 (18p11.1-q11.1), DXZ1 (Xp11.1-q11.1) and DYZ3 (Yp11.1-q11.1).The results were reported following the International System for Human Cytogenomic Nomenclature (ISCN) 2020 [13].

### 2.3. Microarray Experiment

Microarray-based comparative genomic hybridization (aCGH) was performed with the CytoScan™ HD kit (ThermoFisher Scientific, Waltham, MA, USA) using genomic DNA from peripheral blood, following the manufacturer’s instructions. Data were analyzed with the Chromosome Analysis Suite (ChAS) software (ThermoFisher Scientific, Massachusetts, USA).

## 3. Results

### 3.1. Clinical Study

**Figure 1:**
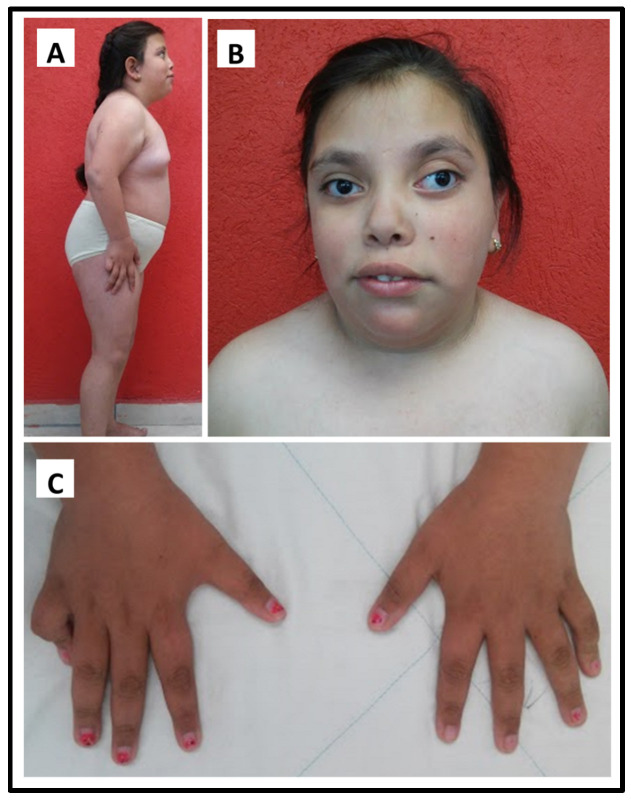
The phenotype of the proband: (**A**) Habitus: short stature, obesity, short neck, rhizomelic shortening of the limbs, hyperlordosis, and limited extension of the elbow. (**B**) Facies: mild frontal bossing, broad nasal bridge, anteverted nostrils, severe nystagmus, divergent left-eye strabismus, low-set ears, short nasal columella, full lips. (**C**) Bilateral fifth finger camptodactyly.

The proband is female, who was 7years old, when first evaluated by the Genetics department, with short stature (percentile < 3, Z-score −2.47) and obesity (BMI 97th percentile). On physical examination, we found an unusual face with mild frontal bossing, broad nasal bridge, anteverted nostrils, severe nystagmus, divergent left-eye strabismus, low-set ears, short nasal columella, full lips, and a high-arched palate. Short neck, cubitus valgus, bilateral fifth finger camptodactyly, broad chest, wide-spaced nipples, multiple nevi, rhizomelic shortening of the limbs, hyperlordosis, and limited extension of both elbows (Figure 1). X-ray showed delayed bone growth. Upon further examination, primary hypothyroidism, hypertriglyceridemia, and hyperinsulinism were diagnosed. The cognitive evaluation demonstrated intellectual disability (ID). At 12 years old pelvic sonogram revealed a hypoplastic uterus and no identifiable ovaries. Type 2 Diabetes Mellitus and hypergonadotropic hypogonadism were diagnosed at 14 years old.

### 3.2. Cytogenetic Study

**Figure 2:**
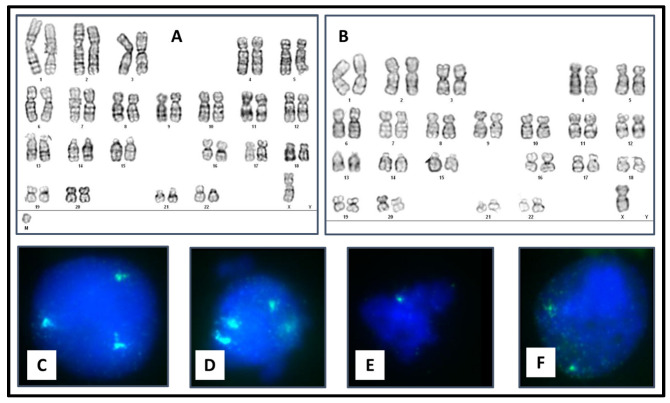
Cytogenetic findings in the proband from peripheral blood (PB) and buccal mucosa (BM). (**A**) PB karyotype 46,X,+mar. (**B**) PB Karyotype 45,X. (**C**) PB FISH with two blue signals (D18Z1) and a green one (DXZ1). (**D**) PB FISH with two blue signals (D18Z1) and two green signals (DXZ1). (**E**) BM FISH with a single green signal (DXZ1). (**F**) BM FISH with two green signals (DXZ1).

Peripheral blood karyotype was performed in 20 metaphases revealing mosaicism of two cell lines. 20% of the cells presented monosomy of the X chromosome, and 80% showed a small marker chromosome. Karyotype result was mos 46,X,+mar[16]/45,X[4] (Figure 2) as a reference,marker was smaller in size than chromosome 20.

Interphase FISH was performed in two different tissues, peripheral blood, and buccal mucosa, with probes for centromeric DNA sequences DXZ1(Xp11.1-q11.1) and DYZ3 (Yp11.1-q11.1) to determine the origin. The results were nucish (DXZ1)×2[57]/(DXZ1)×1[143] for peripheral blood, and nucish (DXZ1)×1[49]/(DXZ1)×2[102]. The percentage of cells with one DXZ1 signal varied between buccal mucosa (32.6%) and peripheral blood (71.5%). No signals of Y chromosome material were present in either tissue. TheaCGH analysis revealed the sSMC content and breakpoints on both arms of the X chromosome. The lengthwas 25.34 Mb, with double terminal deletion, 56.85Mb deletion of Xp (Xpter-Xp11.21) and 73.08 Mb deletion of Xq (Xq21.1-Xqter). Following ISCN 2020, the microarray karyotype could be written as arr[GRCh37] Xp22.33p11.21(1_56850940)×1, Xq21.1(82192264_156040895)×1, Xp11.21q21.1(56850941_82192263)×1.5. The marker chromosome harbors 110 OMIM genes.

## 4. Discussion

**Figure 3:**
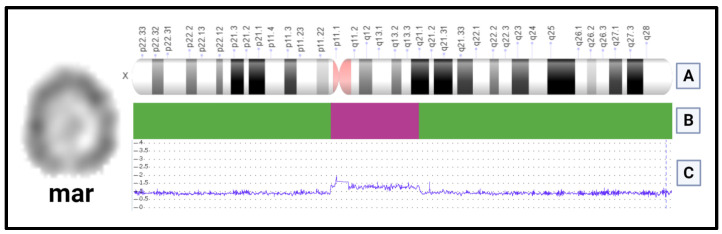
Diagram representing the breaking points in the sSMCT. On the left is the marker chromosome. (**A**) X chromosome ideogram. Provided by NCBI Genome decoration page. (**B**) The magenta bar represents the sSMCT, and the green bars the double terminal deletion. (**C**) Result of the aCGHarr[GRCh37] Xp22.33p11.21(1_5685094056687922)×1, Xq21.1(8326897882192264_156040895)×1, Xp11.21q21.1(56850941_8219226356687923_83268977)×1.5.

The cytogenomic study was performed on this patient because of facial dysmorphism, short stature, limited extension of the elbows, and ID resulting in the finding of an X-derivedsSMCT with double terminal deletion (Xpter-Xp11.21 and Xq21.1-Xqter) most probably in a ring disposition [3,4,5,6]. Furthermore, according to the breaking points provided by the aCGH, the sSMCT present in our patient (Xp11.21q21.1) includes the *XIST locus* (Xq13.2), even though its functional status was not determined. This finding suggests that the unusual phenotype might be explained by improper inactivation of the X-derived ring (Figure 3).

X chromosome inactivation (XCI) is a stochastic phenomenon that compensates for gene dosage imbalance between sex chromosomes and results in the silence of one X chromosome in females by the long non-coding RNA XIST. Once XIST expresses, it coats the future inactive X and starts a cascade of epigenetic events that change the configuration of this chromosome into transcriptional inactive heterochromatin [14]. If this process is not carefully controlled, disease and developmental disorders might result from the increased X-linked gene expression [15,16,17].

Lack of *XIST locus* in X-derived sSMCT and small ring chromosomes (r(X)) has been associated with ID or DD in TS patients and a severe phenotype produced by functional disomy for the genes contained in the marker or ring [18,19]. Kushnick reported in 1987 two TS patients with neurological abnormalities and unusual findings such as facial dysmorphism (“coarse facial features”), strabismus, and syndactyly [20]. Lindgren presented a case series in 1992 of patients with mosaicism with 45,X and marker chromosomes. One of them (patient 2) exhibited clinical similarities to our proband, TS stigmata, DD, and facial dysmorphism, as well as a ventricular septal defect, *foramen ovale,* and patent *ductus ateriosus*, unusual cardiopathies for TS, not present in our patient [21]. Cole presented in 1994 a TS patient who also exhibited ID and a distinctive facial appearance [22]. Mosaicism with monosomy X and r(X) was present in all these cases. Nevertheless, neither described the *XIST* presence or functional state nor the breaking points of the ring chromosomes.

The absence of *XIST locus* and average intellect or development in TS patients had been described in small r(X) with breaking points near the centromere (p11-11.23q11-13.1), as the one reported by Robson in 1994 with left arm asymmetry [23], two cases presented by Turner in 2000 with Kabuki facies [24], and one patient with alopecia *universalis* reported by Bouayed in 2004 [25]. However, Liehr included a case in the sSMC database with r(X)(p11.2q13.1); the ring was reported 54.09–67.79 MB in size by aCGH[9]. This patient presented ID, thus, highlighting how complex it is to establish a direct karyotype-phenotype relationship in TS patients.

In cases with larger r(X) that failed to be inactivated because of the absence of *XIST*, ID was present in TS, accompanied by facial dysmorphism [24], hyperflexible joints [26], strabismus, and scoliosis [27]. In addition, Migeon reported in 1996 a TS patient with profound ID, craniofacial asymmetry, facial dysmorphism, contractures of the elbow, and seizures. The karyotype of this patient (mos 45,X/46,X,del(X)(q21.3-qter)/46X,+r(X)(p22.3q13.2) included maternal isodisomy and mosaicism of a deleted X chromosome with an intact but dysfunctional *XIST locus,* and a small r(X) lacking *XIST* [28]. Table 1 summarizes previous cases reported with an sSMCT and defined ring shape in TS patients with unusual clinical findings.

When sSMC^T^ and r(X) retained the *XIST* gene, variable breakpoints in the short arm of the X chromosome did not seem to be related to the inability to express *XIST.* Tomkins reported the case of a TS patient with DD, dysmorphic facial appearance, syndactyly, and brachydactyly. Cytogenetical analysis showed a small r(X) (p11.3q13) with an intact but dysfunctional *XIST* due to a variant in the gene promoter. Functional disomy of the genes contained in the small ring was presumed to account for the distinctive phenotype in this patient [29]. Kalkan described a TS patient with facial dysmorphism and mild ID in 2006, an sSMC^T^, breaking points in Xp11.3 and Xq13.2, *XIST,* and androgen receptor *(AR) loci* present, without describing its functional status [30]. Liehr has reported four cases with ring-shaped sSMCT, ID/DD, presence of *XIST,* and breakpoints ranging from Xp11.1-11.3 and Xq13-13.3 [7,9]. The breakpoint in the long arm of the X chromosome, when distal to the *XIST locus,* does not seem to be related to the inability to express it adequately, as it may require distal elements to function properly.

sSMC^T^ in ring disposition with breaking points similar to the ones present in our patient (Xp11.2q21.1) have been previously reported with a variable degree of manifestations, from mild manifestations like average intelligence without another dysmorphism [31], ID or DD [9], to severe ones, with microcephaly, facial dysmorphism as frontal bossing, hypertelorism, cup ears, upturned nostrils, microcephaly, brachydactyly, and profound ID/DD [32]. Other clinical findings in patients with r(X) are seizures and contractures of the limbs [18]. Kubota reported two patients with a severe phenotype that retained the XIST locus while exhibiting functional X disomy for the genes present in the r(X)(Xp11.2q21.1) [32]. The increased presence of ID/DD and absence or dysfunction of XIST in TS with r(X) may reflect the bias of ascertainment of patients with severe phenotype.

Even though *XIST* is known as the master regulator of the XCI, the precise mechanism through which it initiates this process remains unknown, features such as genomic distance from the *XIST* locus, gene density, and proximity to long interspersed nuclear elements (LINEs), which act as waystations to enhance XIST RNA coating throughout the Xi 3D space interacts in complex ways that are still under investigation [14]. Although it is not possible to establish a direct relationship between the presence of r(X) in TS and abnormal phenotype, the similarities between the cases that retained *XIST* suggest these rings are not correctly inactivated. It has been proposed that XIST expression is not fully functional in these cases, resulting in incomplete XCI [18].

Another possibility is that RNA is present but not binding correctly and sufficiently to the r(X) [18]. When the X chromosome presents structural defects, it may fail to undergo chromatin conformation changes associated with normal XCI, despite apparently regular *XIST* expression, as it might be occurring in the ring-shaped sSMCT found in this case. We propose that functional disomy of the genes contained in the 25.34Mb sSMCT, as well as the variable degree of mosaicism between tissues containing the marker, are responsible for the ID, facial dysmorphism, and severe phenotype present in our patient.

The limitations to applying these hypotheses to our case are (1) the lack of knowledge of the functional status of *XIST* and (2) an Unknown degree of mosaicism in tissues other than peripheral blood and buccal mucosa. Chromosomal mosaicism in TS is considered a potential modifier of the phenotype, differing unpredictably between the same individual [2,33,34]. sSMC^T^ derived from the X chromosome, as in the case of the patient we report, play a significant role in modifying the classical phenotypic findings. In this case, the proportion of cells with potential functional X disomy varies from 28 to 80%, depending on the analyzed tissue and the used technique. It is possible that different degrees of functional X disomy during critical stages impacted the neurodevelopment of our patient [2,7,10,11,20,22,33,34].

**Table 1:** Clinical comparison of patients with an sSMC^T^ or small ring derived from the X chromosome.

Karyotype	Cells with 45,X (%)	Short Stature	Ovarian Failure	ID/DD	*XIST* Present	Unusual Findings for TS	Reference
mos 46,X,+mar(X)(p11.21q21.1)/45,X	20	+	+	+	+	Facial dysmorphism Divergent strabismusLimited extension of both elbows	Present case
mos 45,X/46,X,r(X)	88	+	+	+	N.A.	Facial dysmorphism. Divergent strabismusLimb contractures Syndactyly	Kushnick (1987) [20]
mos 45,X/46,X,r(X)	42	+	N.A.	+	N.A.	Facial dysmorphism Ventricular septal defect *Foramen ovale* Patent *ductus arteriosus*	Lindgren (1992) [21]
mos 45,X/46,X,r(X)	82	+	N.A.	+	N.A	Facial dysmorphism	Cole (1994) [22]
mos 45,X/46,X,r(X)(p11.23q11.2)/ 46,X,dic(X)(p11)	82	+	+	−	−	Asymmetry of the left arm	Robson (1994) [23]
mos 45,X/46,X,r(X)(p21q22)/ 46,X,min(X)(p11.1q11.1)	40	+	N.A.	+	+ −	Hyper flexible joints	Wolff (1994) [26]
mos 45,X/46,X,r(X)/ 47,X,r1(X),+r2(X)	N.A.	+	N.A.	+	− +	Strabismus Scoliosis	Cantú (1995) [27]
mos 45,X/46,X,del(X)(q21.3-qter)/46X,r(X)(p22.3q13.2)	80	+	+	+	+	Cranial and facial asymmetry with dysmorphism Contractures of the elbow Seizures Maternal iso UPD	Migeon (1996) [28]
mos 45,X/46,X,r(X)(p21.2q13.2)	83	+	+	+	−	Facial dysmorphism	Turner (2000) [24]
mos 45,X/r(X)(p11.22q11.2)	12	+	N.A.	N.A.	−	Kabuki-like *facies*	Turner (2000) [24]
mos 45,X/r(X)(p11q11)	70	+	+	−	−	Kabuki-like *facies*	Turner (2000) [24]
mos 45,X/46,X,r(X)(p11q21.1)	13	+	N.A.	+	+	Facial dysmorphism Microcephaly	Kubota (2002) [32]
mos 45,X/46,X,r(X)(p11q21.1)	10	+	N.A.	+	+	Facial dysmorphism Brachydactyly	Kubota (2002) [32]
mos 45,X/46,X,r(X)(p11.3q13)	28	+	N.A.	+	+	Facial dysmorphism Syndactyly Brachydactyly	Tomkins (2002) [29]
mos 45,X/r(X)(p11q11)	10	+	−	−	−	Alopecia *universalis*	BouayedAbdelmoula (2004) [25]
r(X)(p11.3~11.4q13.3)[19%]/ r(X;X)(p11.3~11.4q13.3::p11.3~11.4q13.3)[2%]	79	+	N.A.	+	+	N.A	Liehr (2007) [7]
r(X)(p11.3q12) aCGH 44.19–64.59 MB	80	+	N.A.	+	+	N.A	Liehr (2007) [7]
mos 45,X/46,X+mar(X)(q11.11q13.2)	95	+	N.A.	+	+	Facial dysmorphism	Kalkan (2016) [30]
mos 45,X/46,X,r(X)(p22.11q21.32)	50	+	+	−	+	−	Chauhan (2016)
mos 45,X/46,X,r(X)(p11.23q21.1)	57	+	N.A.	−	+	N.A.	Li (2020) [31]
r(X)(p11.22q13.1) aCGH 54.09–67.73 MB	43	N.A.	+	+	−	N.A.	Liehr (2023) [9]
r(X)(p11.2?2q21~22)	66	+	+	+	N.A.	N.A.	Liehr (2023) [9]
r(X)(p11.22q22.3)	43	+	+	+	+	N.A.	Liehr (2023) [9]
r(X)(p11.21q13)	36	+	+	+	+	N.A.	Liehr (2023) [9]
r(X)(p1?1.2q13.?3)	77	+	+	+	+	N.A.	Liehr (2023) [9]

Modified from Liehr (2023) “sSMC(X) with defined ring shape” [9] ID: Intellectual disability. DD: Developmental delay. Phenotype present (+), or absent (−). N.A.: Not available.

## 5. Conclusions

The presence of sSMC^T^ influences the clinical manifestations of TS, often with severe phenotypes resulting from the genetic imbalance of X chromosome material due to failure of XCI. In addition, diverse aspects of the sSMC^T^ or r(X) as the size, implicated genes, degree of mosaicism, and the inactivation status of the X chromosome, impact the presence of a distinct phenotype and comorbidities. Therefore, a multidisciplinary team should comprehensively study patients whose karyotypes present these structural abnormalities. Using techniques that identify CNV, as an aCGH assay, allows the accurate identification of chromosomic segments that cannot be recognized with classic Cytogenetics, improving the understanding of the chromosomal composition of the patient. Further studies of the XCI in the structurally abnormal chromosome and the characterization of cases where such anomalies are present are needed to widen our understanding of the clinical diversity of TS patients.

## Data Availability

Not applicable.
